# The Antimicrobial Properties of Modified Pharmaceutical Bentonite with Zinc and Copper

**DOI:** 10.3390/pharmaceutics13081190

**Published:** 2021-08-02

**Authors:** Fotini Martsouka, Konstantinos Papagiannopoulos, Sophia Hatziantoniou, Martin Barlog, Giorgos Lagiopoulos, Triantafyllos Tatoulis, Athanasia G. Tekerlekopoulou, Paraskevi Lampropoulou, Dimitrios Papoulis

**Affiliations:** 1 Department of Geology, University of Patras, 26504 Patras, Greece; geo13118@upnet.gr (K.P.); p.lampropoulou@upatras.gr (P.L.); papoulis@upatras.gr (D.P.); 2 Department of Pharmacy, University of Patras, 26504 Patras, Greece; sohatzi@upatras.gr; 3 Institute of Inorganic Chemistry, Slovak Academy of Sciences (SAS), Dúbravská Cesta 9, 845 36 Bratislava, Slovakia; martin.barlog@savba.sk; 4 Microbiology Department, Quality Assurance and Control Systems—QACS Labs, Antigonis 1, Metamorfosis, 14451 Athens, Greece; geolag@qacs.gr; 5 Department of Environmental Engineering, University of Patras, 2 G. Seferi Str., 30100 Agrinio, Greece; ttatoulis@upatras.gr (T.T.); atekerle@upatras.gr (A.G.T.)

**Keywords:** antimicrobial protection, bentonite, copper, topical formulation, zinc

## Abstract

Pharmaceutical grade bentonite, containing a high amount of montmorillonite, enriched with zinc (Zn) or copper (Cu) (ZnBent and CuBent, respectively) was used as the main component for the creation of formulations for cutaneous use and tested for their antimicrobial capacity. Bentonite (Bent) with added phenoxyethanol (PH) as a preservative and unmodified bentonite were used as control groups. The mineralogical composition, structural state, and physical or chemical properties, before and after the modification of the samples, were characterized utilizing X-ray Diffraction Analysis (XRD), Fourier-Transform Infrared Spectroscopy (FTIR) and X-ray Fluorescence (XRF) techniques, and Scanning Electron Microscope-Energy Dispersive Spectroscopy (SEM, SEM-EDS) analyses. In addition, the profile of zinc and copper concentration from two types of surfaces ZnBent and CuBent, and into Phosphate-Buffered Saline (PBS) are discussed. Finally, the formulations in the form of basic pastes were challenged against bacteria, molds, and yeasts, and their performance was evaluated based on the European Pharmacopeia criteria. The Cu-modified bentonite performed excellently against bacteria and yeasts, while the Zn-modified bentonite only showed great results against yeasts. Therefore, Cu-modified bentonite formulations could offer antimicrobial protection without the use of preservatives.

## 1. Introduction

Clay minerals offer a great alternative for a vast variety of applications due to their unique physical (small particle size and shape, high specific surface area, color, and texture), chemical (low or null toxicity) [1], and physicochemical properties (high ion exchange capacity, sorptive capacity) [2,3,4]. Specifically, they are used as topical semi-solid products in the form of paste and poultices in the pharmaceutical and cosmetic industries. Before considering their use for pharmaceutical—cosmetic preparations, certain pharmaceutical tests (challenge test) should be performed, the results of which must meet the strict chemical, physical and toxicological standards laid down by the European Pharmacopoeia or the United States Pharmacopoeia [4,5]. Many of the chemicals (parabens) used intensively in pharmaceuticals and cosmetics preparations can be durable, bioactive, have a potential for accumulation in the organism, as well as causing endocrine disorders [6,7,8,9]. Although the acute toxicity of these chemicals is considered low, some of them cause serious environmental impacts [10]. In cosmetology, clay minerals are used as protection from sunlight, in creams, powders, and emulsions [11]. The use of clay minerals in medicine is due to their astringent and hemostatic action as well as the treatment of skin diseases [1,12]. In the field of dermatology and cosmetics, their use is equally important and due to the ingredients they contain, they prove to be beneficial against skin lesions in various parts of the body [1,13]. In addition, they act locally as antiseptics [14], disinfectants [14] anti-inflammatory [14,15,16], as dermatological protections [14,15,17], as well as local anesthetics [13,18,19]. They have been used as carriers and have great interest, especially when they are combined with metals known for their antimicrobial and antibacterial properties [19,20,21]. Bentonites are vastly used in the pharmaceutical and cosmetics industry, due to their healing and cosmetic properties [4]. They are generally used in pharmaceutical and cosmetic applications for their indirect antibacterial activity, controlled mainly by their very high cation exchange capacity, high surface area, high swelling capacity, high water dispersibility, high absorption capacity, and non-toxic properties [13,22,23,24]. Bentonite consists chiefly of crystalline clay minerals belonging to the smectite group [25]. The structure of the smectites is 2:1 and consists of a central octahedral sheet of aluminum located between two tetrahedral sheets of silicon dioxide. They have an oily appearance, odorless, soft texture, small particle size (0.2–2 μm), high plasticity and viscosity, similar to skin pH, high swelling capacity high exchange capacity cations (100–200 meq/100 g) [4,26,27,28,29,30,31,32,33,34,35,36,37,38,39] and color ranging from yellow, pink, and gray [4,40].

Systemic antimicrobials are widely used to treat the vast majority of skin infections [40] with the potential for systemic side-effects such as diarrhea and abdominal cramping, while also increasing the antimicrobial resistance within intestinal microflora [41]. The use of topical agents neglects such risks and adds certain advantages such as providing a higher concentration of antibiotics to the affected area and lowering the quantity of active ingredients needed [40]. Copper and zinc, are metals that have been used to create alginate fibers [42,43] or other cosmetic and pharmaceutical formulations [44,45,46,47] and are known for their anti-microbial and inhibitory action on microorganisms, and their help in tissue regeneration without causing cytotoxicity problems in the human body. Various studies have been performed to examine the antimicrobial activities of bentonite or silver-modified bentonite [48,49,50]. However, due to the high cost of silver [50,51], its high cytotoxicity [52,53,54], and the problem of 'argyria' after continuous use of silver [55], new metals may be considered. Recently, Cu-loaded kaolinite showed excellent preservation activity against bacteria and adequate population reduction for molds, while Zn-loaded talc demonstrated significant antimicrobial protection on yeasts [56]. Modified bentonite with metals like zinc or copper, which do not display the above-mentioned negative properties of silver, has not been tested until now for the evaluation of antimicrobial protection in topical use. The combined antimicrobial properties of bentonites and metals (zinc, copper), the low risk of using them, as well as and the abundance of such materials in the markets motivate our research team to conduct this research. The purpose was to determine the suitability of using pharmaceutical-grade bentonites in topical administration in paste form by enriching them with copper and zinc to develop the best possible antimicrobial properties (Supplementary Materials).

## 2. Material and Methods

### 2.1. Materials

Pharmaceutical-grade bentonite was purchased from CHEMCO by Syndesmos S.A. (Athens, Greece). The zinc (ZnCl_2_) and copper (CuCl_2_) precursors were purchased from Chem-Lab (Zedelgem, Belgium).

### 2.2. Samples Preparation

To load the clay particles with Zn or Cu ions, the samples were saturated with 1 M ZnCl_2_ and CuCl_2_ solutions respectively. The solutions were prepared by mixing the molar mass of the chlorides with 1 L of distilled water [57].

The bentonite sample and the solution were mixed (ratio of 10 g/150 mL), stirred for 10 min, and then the mixture was centrifuged. The separated chloride solution was disposed of and refilled with the chloride solutions. This procedure was repeated five times [57] and the samples were washed with distilled water to remove excess chloride. Finally, each sample was dried and pulverized to powder. The samples obtained were labeled as CuBent (Cu-bentonite) and ZnBent (Zn-bentonite). Also, the unmodified bentonite was labeled as Bent.

The preparation of the pastes included the mixing of clay powders (25% *w*/*w*), with starch (25% *w*/*w*), glycerin (5% *w*/*w*), and deionized water (45% *w*/*w*) and then stirring until total homogeneity. Consequently, pastes containing unmodified bentonite powder (Bentp), modified-clay powders (ZnBentp, CuBentp), and unmodified bentonite with added phenoxyethanol (PHBentp) were created. The PHBentp sample was created by replacing 1% *w*/*w* of water with 1% *w*/*w* phenoxyethanol that served as the preservative. Each paste weighed 120 g [58,59,60,61].

### 2.3. Characterization

#### 2.3.1. X-ray Diffraction Analysis

The X-ray diffraction technique was used to determine the mineralogical composition of the samples. A Bruker D8 advance diffractometer (Bruker, Billerica, MA, USA) with nickel-filtered copper Kα radiation was used complemented by DIFFRAC plus EVA12^®^ software (Bruker-AXS) [62] based on ICDD (International Centre for Diffraction Data) Powder Diffraction File of PDF-2 2006. The XRD patterns were obtained between 2^o^ and 60° at a scanning rate of 2°/min. The samples were prepared by the dropper method. Semi-quantitative analyses were performed by TOPAS 3.0^®^ software (TOPAS MC Inc., Oakland, CA, USA), based on the Rietveld method refinement routine as well as by the peak area method using the DIFFRAC plus EVA12^®^ software and its “Area” tool. The errors of each phase were estimated under 1%.

#### 2.3.2. Scanning Electron Microscope-Energy Dispersive Spectroscopy

Images revealing the surface morphology of the clay particles were obtained by using a scanning electron microscope SEM JEOL 6300 (JEOL, Tokyo, Japan) operating at 30 kV with an energy dispersive spectrometer (EDS). By utilizing energy dispersive spectroscopy, microelemental analysis was conducted and the presence of the metals was represented by pseudocolorization. Carbon coating under vacuum on all samples was used for the best efficiency.

#### 2.3.3. Fourier-Transform Infrared Spectroscopy

Any structural change after the chemical modification was investigated using infrared spectra obtained on a Nicolet 6700 Fourier Transform Infrared (FTIR) spectrometer from Thermo Scientific™, (Thermo Scientific, Waltham, MA, USA) while IR source (wolfram wire), KBr beamsplitter, and DTGS detector were used for measurement in the middle IR region (MIR 4000–400 cm^−1^). The KBr pellet press technique (0.5–1 mg of sample homogenized with 200 mg KBr) was also used to collect MIR transmission spectra. The minimization of adsorbed water was achieved by heating the pellets overnight at 140 °C. For each sample, 128 scans with a resolution of 4 cm^−1^ were recorded.

#### 2.3.4. X-ray Fluorescence

The elemental analysis of the samples was conducted by X-ray fluorescence (XRF) diffusion using a ZSX PRIMUS II, from Rigaku (Tokyo, Japan), with an elemental range of Be to U.

#### 2.3.5. Profile of Metals Concentration

In addition, zinc and copper profile concentrations for both types of surfaces ZnBent and CuBent into PBS from Sigma-Aldrich (Darmstadt, Germany) were determined. Four different times were examined (t_0_ = 5 min, t_1_ = 30 min, t_2_ = 60 min, and t_3_ = 120 min), while 5 mL of phosphate-buffered saline (PBS) was added each time. Phosphate-buffered saline (PBS) is a buffer that is often used in biological research and studies because its concentrations of ions and osmotic molecules match those of microorganisms. It is a saline isotonic solution containing sodium phosphate, sodium chloride, and in some cases potassium chloride and potassium phosphate. Specifically, one PBS buffer tablet was dissolved in 200 mL of deionized water to give 0.01 M phosphate buffer, 0.0027 M potassium chloride, and 0.137 M sodium chloride, pH 7.4, at 25 °C (Sigma-Aldrich, Merck KGaA, Darmstadt, Germany). Specifically, four different falcons were made for each surface tested (ZnBent or CuBent), each falcon containing 0.25 g of material. 5 mL of phosphate-buffered saline (PBS) was then added to each of the eight falcons. PBS was in contact with the material for four different reaction times (t_0_ = 5 min, t_1_ = 30 min, t_2_ = 60 min, and t_3_ = 120 min), with one falcon of each material corresponding to one contact time. The samples for homogenization were then stirred with the Vortex laboratory stirrer for 30 seconds and then centrifuged at 3000 rpm and then the solid was separated from the supernatant (liquid) by a pipette and collected in falcons. The procedure for each surface was repeated three times (triplicate) to create zinc and copper release curves of ZnBent and CuBent samples.

Subsequently, a specific quantity of samples ZnBent and CuBent were withdrawn in 5, 30, 60, and 120 min while nitric acid (2%) was used for metal dissolution and stabilization for ICP-MS analysis. Zinc and copper concentrations were determined using the Optima 8000ICP-OES (Perkin Elmer, Inc., Waltham, MA, USA) instrument that specializes in the determination of metals and trace elements. Argon was used with the ICP-OES system while nitrogen for the optical purge gas. The wavelength used for Zn and Cu was 213.855 and 324.747 nm, respectively, while the plasma view for the metals was axial for both metals. The experimental conditions used were: Plasma gas flow: 15 L/min, Auxiliary gas flow: 0.2 L/min, Nebulizer gas flow: 0.55 L/min, RF power: 1300 Watts, Purge flow: Normal, Peristaltic pump flow rate: 1.5 mL/min. The average values and standard deviations were estimated by three independent experiments Axis X: Exposure time (min), Axis Y: Concentration (Cu/Zn mg L^−1^).

### 2.4. Statistical Analysis

The experimental sets were conducted in triplicate and the results were expressed as mean ± standard deviation (SD) of measurements. Mean values were obtained from three samples taken from three independent bioreactors characterized by the same operating conditions. Statistically significant differences between data were evaluated using Student’s *t*-test (Microsoft Office Excel 2007, Redmond, WA, USA) confidence interval for 95% probability, and a value of *p* ≤ 5% was considered statistically significant.

### 2.5. Evaluation of Antimicrobial Protection

The Preservation Efficacy Test (PET), also known as the Challenge test, has been used for the determination of each formula’s antimicrobial effectiveness. PET is a reference method, primarily designed for multiuse water-soluble or water-miscible products, during which, the formulation is challenged by several specified micro-organisms (i.e., bacteria, yeasts, and molds), to evaluate if the formulation is adequately preserved. More specifically, the test consists of challenging the preparation under test, with a prescribed inoculum of suitable micro-organisms, storing the inoculated preparation at 20–25 °C, avoiding sunlight, withdrawing samples from the container at specified intervals of time, and counting the micro-organisms in the samples so removed. The preservative properties of the preparation are adequate if, in the conditions of the test, there is a significant fall or no increase, as appropriate, in the number of micro-organisms in the inoculated preparation after 2, 7, 14, and 28 days. Results are expressed in log reduction values.

## 3. Results and Discussion

### 3.1. X-ray Diffraction Analysis (XRD)

**Figure 1:**
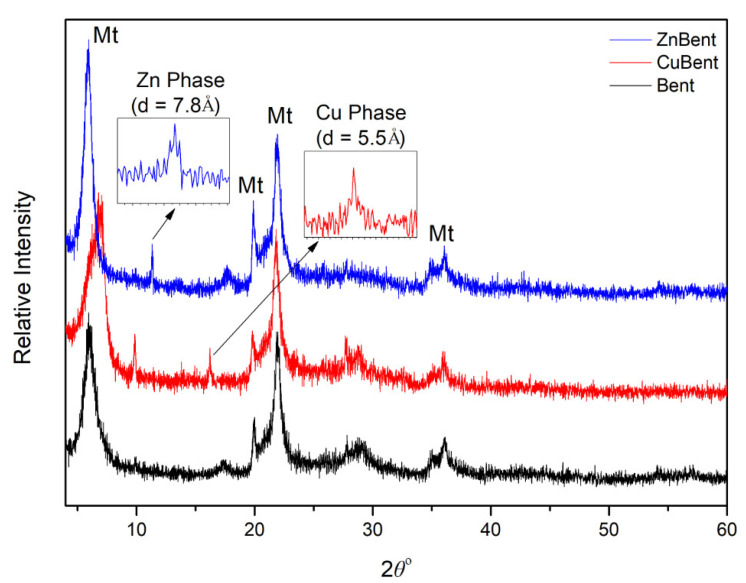
X-ray diffraction patterns of Unmodified Bentonite (Bent), Zinc Modified Bentonite (ZnBent), and Copper Modified Bentonite (CuBent). (Legend: Mt: Montmorillonite, Zn Phase: Simonkolleite syn, Cu Phase: Copper chloride hydroxide).

Figure 1 illustrates the XRD patterns of the unmodified bentonite, Zn modified bentonite, and Cu modified bentonite. The main component of the samples is montmorillonite with its characteristic peaks appearing at 14.48 Å and 4.44 Å, while quartz presence is indicated slightly above detection limits as its two main peaks are slightly visible. Some new peaks are revealed after the modification at 7.8 Å for ZnBent and 5.5 Å for CuBent. This implies that new phases were synthesized during the modification process, possibly salt compounds in the form of oxides in low amounts of 4% (PDF card numbers: 7-155 & 18-0439 for ZnBent and CuBent samples, respectively) as indicated by their relatively low reflections. The absence of other impurities corresponds to the purity expected in pharmaceutical clay.

### 3.2. Fourier-Transform Infrared Spectroscopy (FTIR)

**Figure 2:**
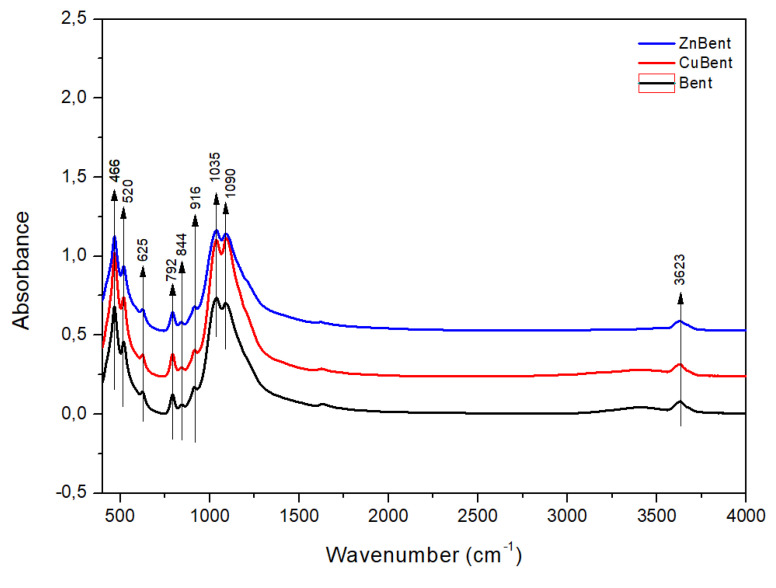
FT-IR spectra diagram of Bent, ZnBent, and CuBent and powder samples.

The MIR spectra of bentonite were measured to obtain information on the composition of initial (Bent) and modified (ZnBent, CuBent) samples. The spectra showed bands characteristic for the dioctahedral smectite with low iron content (Figure 2). The stretching region shows the typically broad band at 3623 cm^−1^ assigned to the vibrations of the OH groups coordinated to octahedral cations (Al_2_OH, AlMgOH, and AlFe^3+^OH) [63]. The complex band in the 1100–1030 cm^−1^ region corresponded to the in-plane Si–O–Si stretching vibrations of montmorillonite at 1035 cm^−1^, stretching vibrations of quartz at 1090 cm^−1^ and from 2nd derivation of spectra (not shown) was observed weak band assigned to Si-O perpendicular vibrations of montmorillonite at 1100 cm^−1^. In the bending region, two peaks at 916 cm^−1^ and 844 cm^−1^ are related to δ (Al_2_OH) and δ (AlMgOH). The Al–O–Si bending band appeared at 520 cm^−1^ and Si–O–Si bending at 466 cm^−1^ [63]. The peak at 625 cm^−1^ was assigned to coupled Al–O out-of-plane and Si–O bending vibrations. The quartz admixture was also found in the examined samples at 792 cm^−1^. By comparison of the positions of the bands, no significant differences between samples were found.

### 3.3. Surface Characterizations

#### Scanning Electron Microscope-SEM-Energy Dispersive Spectroscopy (SEM-EDS)

**Figure 3:**
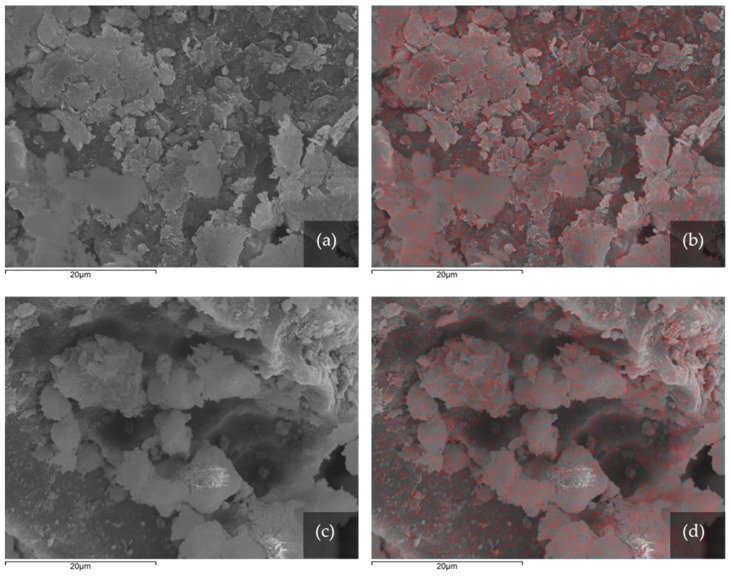
SEM photomicrographs showing before (**left**) and after (**right**) elemental mapping of (**a**,**b**) ZnBent and (**c**,**d**) CuBent, respectively. The distribution of zinc and copper, respectively, is indicated by red dots.

As shown in Figure 3, montmorillonite is in the form of thick flakes exhibiting irregular and curling edges [64]. The clay particles are usually found as particle aggregates and not as single particles. As a result, the particle (mainly particle aggregates) size ranges from 5 μm to 30 μm which is a lot larger than montmorillonite's usual particle size. The distribution of the metal ions is illustrated via elemental mapping utilizing energy dispersive X-ray spectrometry. As seen in Figure 3, the metal ions are distributed homogenously and densely upon the smectite crystals. This is the desired result, as microbial growth could occur in large areas where metal ions are scarce or absent. Moreover, this distribution validates the theory that the metals have not formed distinguishable aggregates, which would be susceptible to mechanical removal, thus downgrading the antimicrobial potential of the material.

### 3.4. Chemical Characterization

#### X-ray Fluorescence Spectroscopy Analysis (XRF)

Table 1 shows the trace elements present in each sample. The modified bentonite is richer in copper and zinc for CuBent and ZnBent respectively in comparison to the unmodified bentonite (Table 2).

### 3.5. Profile of Metal Concentration

**Figure 4:**
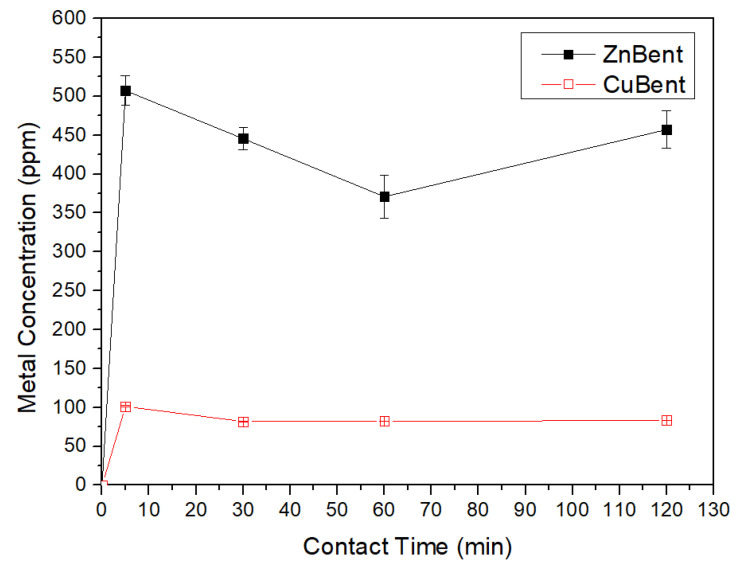
Concentrations of Ζn and Cu in PBS in relation to contact time.

The concentration of Zn and Cu ions released from ZnBent and CuBent respectively in the PBS is presented in Figure 4. The concentrations of both metals elevated rapidly to a maximum value of 500 ppm (zinc) and 100 ppm (copper) after 5 min of contact time. Regarding Zn, the concentration of zinc ions was decreased until reaching the minimum value of 370 ppm at 60 min and then was increased again up to the value of 470 ppm at 120 min. Statistically significant differences were reported between the sets of t_0_–t_1_, t_0_–t_2_, t_0_–t_3_, t_1_–t_2_, t_2_–t_3_ (*p* < 0.05), while no difference was noted between set t_1_–t_3_ (*p* = 0.52). Concerning, copper ions their concentration was decreased to 80 ppm at 30 min of contact time and remained at this level until the 120 min mark. The concentration of free copper ions decreases with the formation of oxides, while more copper ions are released from the smectite to compensate for this decrease until an equilibrium is achieved. Statistically significant differences were reported between sets t_0_–t_1_, t_0_–t_2_, t_0_–t_3_, t_1_–t_2_, t_2_–t_3_ (*p* < 0.05), while no difference was noted between set t_1_–t_3_ (*p* = 0.26). Additionally, it is worth noting that the difference between the maximum concentrations of each metal can be attributed to the adsorption preference of smectites between Zn and Cu ions. Sipos et al., (2018) [65], and Baghenejad et al., (2016) [66] refer that Cu is characterized by higher sorption than Zn in soils with smectite clay mineralogy. Specifically, the affinity sequence of the various metals studied by Sipos et al. (2018) [65] is as follows: Pb > Cu > Cd > Zn.

### 3.6. Challenge Test

There are several PET Protocols, like the compendial methods of European and U.S. Pharmacopoeia, or ISO 11930. During this study, an alternative PET Protocol has been used, known as mixed Protocol, which is based on the compendial protocols but is more demanding [56]. The mixed protocol is based on the compendia methods and includes eleven (11) Gram-positive and Gram-negative bacteria, yeast, and molds (Table 3). The selection of the 11 stains is based on spoiling microorganisms usually found on cosmetics production sites and are also representative of the contaminants that are likely to be exposed, during the product's intended use. The contamination level is of the same level with the compendial protocols (10^5^–10^6^ colony-forming units, cfu/g) and the key difference lies in the mixed inoculation. Unlike the Compendial Pharmacopoeia tests and ISO 11930, in which five specified micro-organisms are used as single strain inoculations, the PET mixed protocol includes 11 specified micro-organisms used as grouped (mixed) inocula. The multi-strain contamination simulates the real-life conditions and the increased number of strains challenges in a greater level, the formulation under test. The mixed protocol involves, for each group of test micro-organisms, placing the formulation in contact with calibrated inoculums (10 g of the test formulation, 0.1 mL of calibrated inoculums) and measuring the number of surviving micro-organisms at defined intervals (2nd, 7th, 14th, and 28th day) during a period of 28 days. The containers of inoculation formulation are stored in a dark place at (22.5 ± 2.5 °C).

The enumeration method used was the plate count method-pour plate technique, using 1ml as inoculation quantity. For each time interval and group of micro-organisms, the log reduction value was calculated and compared to the minimum values required for evaluation according to criteria A or B (European Pharmacopoeia, current edition).

The preservative properties of the preparation are adequate if, in the conditions of the test, there is a significant fall or no increase, as indicated by the European Pharmacopoeia criteria. The criteria for the evaluation of PET are given in Table 4 in terms of the log reduction in the number of viable micro-organisms (cfu) against the value obtained for the inoculums. Criteria A expresses the recommended efficacy to be achieved. In justified cases where Criteria A cannot be attained, for example, for reasons of an increased risk of adverse reactions, Criteria B must be satisfied.

The microbial load of all samples was evaluated prior to the challenge test, to determine whether the initial microbial load is low enough for reliable results to be obtained. Any paste that exceeds the limits for total aerobic microbial count, molds, and yeasts given by the European Pharmacopoeia 10.0 is not eligible to proceed to the challenge test. Specifically, all samples passed the sterility test except for the unmodified bentonite paste (Bentp) which developed a total aerobic microbial load >3 × 10^3^ cfu/g five days after its preparation (Table 5). Moreover, this result is corroborated by other authors like Jou et al., (2016) [67] and Holesova et al., (2013) [68] which reported that natural clay minerals show no antibacterial effect. Therefore, the antibacterial properties of clay minerals are attributed to coexisting inorganic species such as Zn [56,69,70] and Cu ions [56,71,72,73].

The total count of bacteria, molds, and yeasts starting from the initial inoculation at the predetermined time intervals are summarized in Table 6, while the logarithmic reduction of CFU and the score for each sample according to the relevant criteria are presented in Table 7. Specifically, CuBentp showed excellent resistance to bacteria and yeasts with similar results to the phenoxyethanol sample, as the total count for bacteria and yeasts was found lower than 100 cfu/g within two days and reduced to a minimum count of <10 cfu/g until the seventh day, thus passing criterion A. Additionally, it demonstrated an adequate reduction of CFU for molds barely missing criterion A with 1.41 instead of 2 log reduction at day 14, overall satisfying criterion B. In this case, the microbiological risk of the product should be assessed during the product development phase, taking into consideration control factors other than the formulation. Such a factor may be the type of container, like an airless container with a pump, that prevents the contact of the product with the environment, providing higher protection, or the strict application of good manufacturing practices during production [74]. The excellent antibacterial behavior of copper-enriched bentonite was also referred to by Das et al., (2014) [75] and Khurana et al., (2015) [76] in their studies.

**Figure 5:**
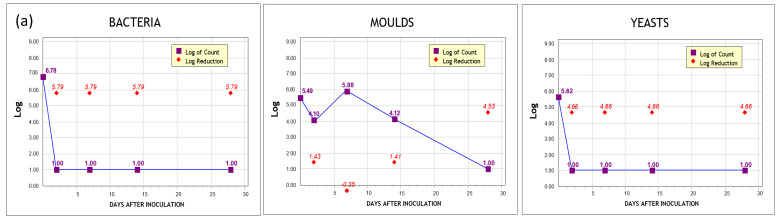

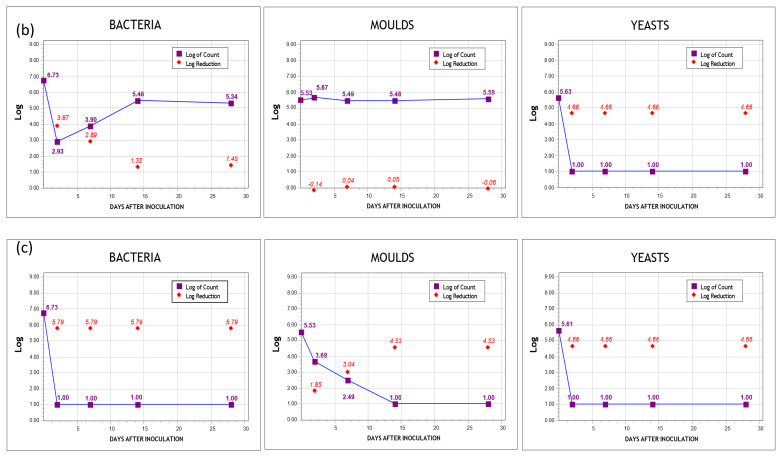
Logarithmic reduction charts of each sample against bacteria, molds, and yeasts (**a**) CuBentp, (**b**) ZnBentp, and (**c**) PHBentp.

Moreover, ZnBentp showed excellent resistance to yeasts, satisfying criterion A, although its performance against molds and yeasts was poor rendering it unable to satisfy any criteria. Finally, the results of the antimicrobial protection for the PHBentp paste were excellent, satisfying criterion A for each microorganism. Total counts for each day paired with the corresponding reduction rates are plotted logarithmically in Figure 5.

## 4. Conclusions

In the present study, the antimicrobial protection of pastes composed of copper or zinc-modified bentonite was evaluated. Firstly, mineralogical analyses via XRD verified the purity of the pharmaceutical bentonite, identifying montmorillonite as its main characteristic phase, while scanning electron microscopy revealed a homogenous dispersion of copper and zinc ions on the main surfaces and edges of clay crystals. Moreover, the abundance of each metal ion was confirmed via chemical analysis (XRF). On the other hand, the structural composition of the samples after the chemical modification was not affected, as no differences were detected between the examined FTIR spectra of modified and unmodified samples. According to the ion release results, the concentrations of both Zn and Cu ions in the PBS peaked within the first 5 min of contact time, thus implying that most ions were released immediately after the samples were submerged into the solution. Overall, the modified pastes have shown excellent results against microbial contamination achieving similar results to the phenoxyethanol control sample. The excess antimicrobial metals that form salts have probably increased the antimicrobial properties of the samples. Specifically, CuBentp demonstrated excellently against bacteria and yeasts satisfying criterion A, while also performing adequately against molds satisfying criterion B. Despite this, controlling certain factors like using airless containers or the strict application of good manufacturing practices can ensure total antimicrobial protection. Contrary, ZnBentp showed no significant antimicrobial protection against bacteria or molds but performed excellently against yeasts satisfying criterion A. Both ingredients could be used in combination with other preservatives that are active against molds, reducing their concentration, or with other natural ingredients with complementary antimicrobial action that are not listed in Annex V of the cosmetic regulation EU 1223/2009. In conclusion, the chemically modified semi-solid bentonite formulations could serve as an alternative solution that offers antimicrobial protection without the use of preservatives, which are responsible for human health and environmental degradation.

## Figures and Tables

**Table 1:** Major element composition (% oxides) (XRF) of Bent, ZnBent, and CuBent powder samples.

Oxide	Bent	ZnBent	CuBent
SiO_2_	77.44	74.53	75.35
Al_2_O_3_	9.87	9.07	9.13
Fe_2_O_3_	0.8	0.69	0.83
MnO	0.01	0.01	0.00
MgO	4.53	3.75	3.78
CaO	0.78	0.3	0.21
Na_2_O	0.87	0.77	0.33
K_2_O	0.8	0.73	0.57
TiO_2_	0.07	0.06	BDL
P_2_O_5_	0.19	0.11	0.02
LOI	4.4	6.97	7.37
Sum	99.76	96.99	97.59

BDL = Below Detection Limit LOI = Loss of ignition.

**Table 2:** Trace element composition (ppm) (XRF) of Bent, ZnBent, and CuBent powder samples.

Element	Bent	ZnBent	CuBent
Cr	7	10	BDL
Co	BDL	BDL	BDL
Cu	BDL	3	45,637
Zn	97	38,266	37
Sr	91	43	42
Y	46	31	136
Hf	6	7	48
Pb	BDL	BDL	26

BDL = Below Detection Limit.

**Table 3:** Types of microorganisms tested.

Test Organisms					
Gram-positive bacteria	*Staphylococcus aureus* ATCC 6538		*Staphylococcus epidermis* ATCC 12228	*Kokuria rhizophilia* ATCC 9341	
Gram-negative bacteria	Enterobacteria		Pseudomonas		
	*Escherichia coli* ATCC 8739	*Enterobacter* *gergoviae* ATCC 33028	*Pseudomonas* *aeruginosa* ATCC 9027	*Burkholderia* *cepacia* In house	*Pseudomonas* *luteola* ATCC 43330
Yeasts	*Candida albicans* ATCC 10231	Molds	*Aspergillus* *brasiliensis* ATCC 16404	*Penicillium* *aurantiogriseum* ATCC 16025	

ATCC (Wesel, Germany).

**Table 4:** European Pharmacopoeia, Criteria A and B, for cutaneous application, minimum reduction in log units.

Criteria A				
Time	2nd Day	7th Day	14th Day	28th Day
Bacteria	2 log	3 log	-	NI
Yeasts	-	-	2 log	NI
Molds	-	-	2 log	NI
**Criteria Β**				
Time	2nd Day	7th Day	14th Day	28th Day
Bacteria	-	-	3 log	NI
Yeasts	-	-	1 log	NI
Molds	-	-	1 log	NI

NI: No increase.

**Table 5:** Initial microbial load test according to the European Pharmacopoeia method.

Paste	Parameter	Result (cfu/g)	Limits (cfu/g)
Bentp	Total Aerobic Microbial Count Molds & Yeasts	>3.0 × 10^3^ <10	<1.0 × 10^2^ <10

**Table 6:** Mixed culture—Preservation efficacy test.

Paste	Parameter	Sterility Control	Inoculation	0 Time	2nd Day	7th Day	14th Day	28th Day
CuBentp	Bacteria	<10	6.2 × 10^6^	6.1 × 10^6^	<100	<10	<10	<10
	Molds	<10	3.4 × 10^5^	3.1 × 10^5^	1.3 × 10^4^	7.6 × 10^5^	1.3 × 10^4^	<10
	Yeasts	<10	4.6 × 10^5^	4.2 × 10^5^	<100	<10	<10	<10
ZnBentp	Bacteria	<10	6.2 × 10^6^	5.4 × 10^6^	8.5 × 10^2^	8.0 × 10^3^	3.0 × 10^5^	2.2 × 10^5^
	Molds	<10	3.4 × 10^5^	3.4 × 10^5^	4.7 × 10^5^	3.1 × 10^5^	3.0 × 10^5^	3.9 × 10^5^
	Yeasts	<10	4.6 × 10^5^	4.3 × 10^5^	<100	<10	<10	<10
PHBentp	Bacteria	<10	6.2 × 10^6^	5.4 × 10^6^	<100	<10	<10	<10
	Molds	<10	3.4 × 10^5^	3.4 × 10^5^	4.8 × 10^3^	3.1 × 10^2^	<10	<10
	Yeasts	<10	4.6 × 10^5^	4.1 × 10^5^	<100	<10	<10	<10

Units: cfu/g.

**Table 7:** Criteria of acceptance. (Evaluation Criteria).

Paste	Log Reduction	2nd Day	7th Day	14th Day	28th Day	Criterion A	Criterion B	Test Result
CuBentp	Bacteria	5.79	5.79	5.79	5.79	✓		Satisfactory
	Molds	1.43	−0.55	1.41	4.53		✓	meet the relevant
	Yeasts	4.66	4.66	4.66	4.66	✓		B-criteria
ZnBentp	Bacteria	3.87	2.89	1.23	1.46			Failed does not
	Molds	−0.14	0.04	0.06	-0.06			meet the relevant
	Yeasts	4.66	4.66	4.66	4.66	✓		A/B-criteria
PHBentp	Bacteria	6.79	6.79	6.79	6.79	✓		Satisfactory
	Molds	1.06	3.04	4.65	4.65	✓		meet the relevant
	Yeasts	4.06	4.06	4.06	4.06	✓		A-criteria

## Data Availability

Data sharing does not apply to this article.

## Supplementary Materials

The following are available online at https://www.mdpi.com/article/10.3390/pharmaceutics13081190/s1, Figure S1. SEM photomicrographs of montmorillonite showing crystals in flakes exhibiting irregular and curling edges in different magnifications 200 nm, 300 nm, 1 μm, and 10 μm, respectively.
