# Supplementary material for: The Antimicrobial Properties of Modified Pharmaceutical Bentonite with Zinc and Copper
Source: Pharmaceutics. 2021 Aug 2;13(8):1190. doi: 10.3390/pharmaceutics13081190 (PMC8399475; doi:10.3390/pharmaceutics13081190)
Supplement: Supplementary file 1 [file pharmaceutics-13-01190-s001.zip › pharmaceutics-1303924-supplementary.pdf]

pharmaceutics

MDPI

Supplementary Materials: The Antimicrobial Properties of Modified Pharmaceutical Bentonite with Zinc and Copper

Fotini Martsouka, Konstantinos Papagiannopoulos, Sophia Hatziantoniou, Martin Barlog, Giorgos Lagiopoulos, Triantafyllos Tatoulis, Athanasia G. Tekerlekopoulou, Paraskevi Lampropoulou and Dimitrios Papoulis

Materials and Methods

Characterization

Scanning Electron Microscope-Energy Dispersive Spectroscopy

Scanning Electron Microscopy (SEM) (Zeiss SUPRA 35VP electron microscope) was used to investigate the surface morphology of the clay particles. The samples were prepared by placing the powders in Au-holders.

Results and Discussion

Surface Characterization

Scanning Electron Microscope- SEM

As shown in Figure S1, montmorillonite is in the form of thick flakes exhibiting irregular and curling edges [1,2]. The clay particles are usually found as particle aggregates and not as single particles. As a result, the particle larger dimension size seems to be reaching 30 μm which is a lot larger than montmorillonite's usual particle size. In any case, this is a dimension that is referred to as particle aggregates rather than single particles.

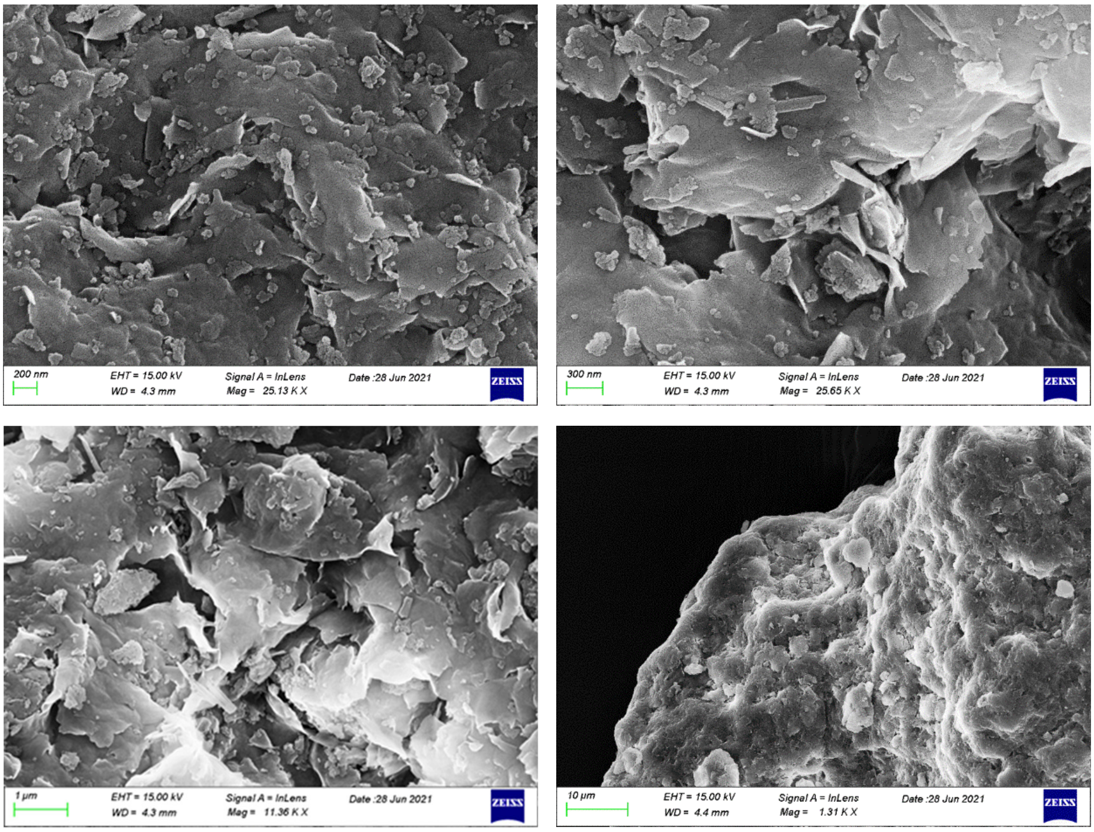

Figure S1. SEM photomicrographs of montmorillonite showing crystals in flakes exhibiting irregular and curling edges in different magnifications 200 nm, 300 nm, 1 μm, and 10 μm, respectively.

References

1. Murray, H.H. Overview - clay mineral applications. Applied Clay Science. 1991, 5(5-6), 379-395; doi: 10.1016/0169-1317(91)90014-Z.
2. Viseras, C., Lopez Galindo, A. (1999). Pharmaceutical applications of some spanish clays (sepiolite, palygorskite, bentonite): some pre formulation studies. Applied Clay Science. 1999, 14, 69– 82; doi: 10.1016/S0169-1317(98)00050-7.
